# Oxidative Stress Defense Module in Lung Cancers: Molecular Pathways and Therapeutic Approaches

**DOI:** 10.3390/antiox14070857

**Published:** 2025-07-13

**Authors:** Eunsun Lee, Jeong Hee Hong

**Affiliations:** Department of Physiology, College of Medicine, Lee Gil Ya Cancer and Diabetes Institute, Gachon University, 155 Getbeolro, Yeonsu-gu, Incheon 21999, Republic of Korea; eunsunpp@gachon.ac.kr

**Keywords:** oxidative stress, redox regulation, oxidative stress defense system, lung cancer

## Abstract

The regulation of oxidative stress is an effective strategy for treating cancers. Therapeutic strategies for modulating an undesirable redox balance against cancers have included the enhancement of oxidative components, reducing the action of antioxidant systems, and the combined application of radiation and redox-modulating drugs. A precise understanding of redox regulation is required to treat different kinds of cancer. This review focuses on the redox regulation and oxidative stress defense systems of lung cancers. Thus, we highlighted several enzymatic antioxidant components, such as superoxide dismutase, catalase, heme oxygenase-1, peroxiredoxin, glutaredoxin, thioredoxin, thioredoxin reductase, glutathione peroxidase, and antioxidant components, including glutathione, nuclear factor erythroid 2–related factor 2, 8-oxo-7,8-dihydro-2′-deoxyguanosine, and mitochondrial citrate carrier SLC25A1, based on PubMed and Scopus-indexed literature. Understanding the oxidative stress defense system in lung cancer would be beneficial for developing and expanding therapeutic strategies, such as drug development, drug design, and advanced delivery platforms.

## 1. Introduction

Oxidative stress plays a critical role in the pathogenesis of various diseases, including cancer, neurodegenerative disorders, metabolic diseases, and cardiovascular diseases [1]. The fundamental antioxidant enzymes are essential for maintaining redox balance under stress conditions and protecting cells against oxidative damage. Thus, cells possess well-organized antioxidant defense systems composed of both enzymatic and non-enzymatic antioxidants [2]. Although a broad array of enzymatic antioxidant systems contribute to cellular redox regulation, in this context, a focused understanding of the cellular antioxidant defense mechanisms—particularly the enzymatic antioxidant systems—has been considered essential for effectively neutralizing reactive oxygen species generated as by-products of metabolic processes [1]. Enzymatic antioxidants are composed of superoxide dismutase (SOD), catalase (CAT), heme oxygenase-1 (HO-1), peroxiredoxin (PRX), glutaredoxin (GRX), glutathione reductase (GR), thioredoxin (TRX), thioredoxin reductase (TrxR), and glutathione peroxidase (GPX) and are extensively reviewed elsewhere [2,3,4].

Although various therapeutic approaches have been developed for cancer treatment, the modulation of the antioxidant defense system has been considered a promising strategy. The lungs are highly susceptible to oxidative damage, such as cigarette smoke, and weak to redox imbalance. Moreover, it is known that lung cancers possess upregulated antioxidant components such as GSH and Nrf2 [5]. Accordingly, in this review, we focus on the role of GSH, Nrf2, and the enzymatic antioxidant defense system in lung cancers, especially non-small-cell lung cancer (NSCLC), and discuss potential combinatorial therapeutic strategies that target antioxidant pathways to enhance anti-lung cancer efficacy. Moreover, the recently identified 8-oxo-7,8-dihydro-2′-deoxyguanosine (8-oxodG) and mitochondrial citrate carrier SLC25A1 with regard to antioxidant components are discussed.

## 2. Methodology

This review was conducted using systematically published literature related to oxidative stress regulation and redox balance in cancer, with a specific focus on lung cancer. Related scientific articles were identified through comprehensive searches on the PubMed and Scopus databases. Search terms included combinations of keywords such as oxidative stress, redox regulation, lung cancer, enzymatic antioxidant systems (SOD, CAT, HO-1, PRX, GRX, TRX, TrxR, and GPX), and redox-associated components (GSH, Nrf2, 8-oxodG, and SLC25A1). Inclusion criteria included studies that discussed the roles of oxidative stress defense systems in cancer, particularly lung cancer. Selected articles were screened based on titles and abstracts. Key findings were extracted and categorized according to antioxidant system components, their redox modulation strategies, and their potential implications for therapeutic development.

## 3. GSH

### 3.1. GSH-Driven Redox Regulation

**Figure 1:**
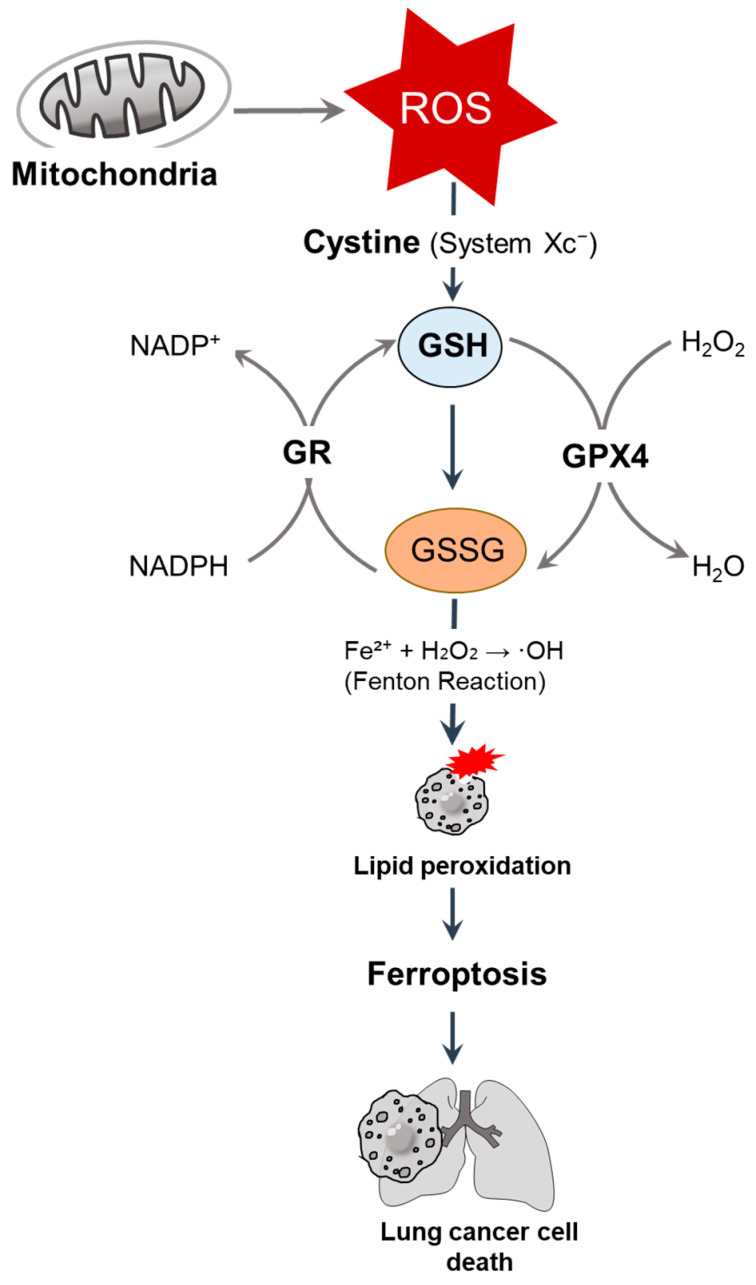
GSH-mediated redox regulation and ferroptosis in lung cancer cells. Mitochondria-derived ROS are neutralized by GSH, the most abundant intracellular antioxidant, and synthesized through cystine uptake via system Xc^−^. GSH serves as a cofactor for GPX4, reducing hydrogen peroxide (H_2_O_2_) and lipid peroxides. Under oxidative stress, GSH is oxidized into GSSG, which is then regenerated by GR in an NADPH-dependent manner [9]. The depletion of GSH promotes the iron-catalyzed Fenton reaction (Fe^2+^ + H_2_O_2_ → ·OH), resulting in lipid peroxidation and inducing ferroptosis [10], ultimately leading to lung cancer cell death. ROS, reactive oxygen species; GSH, glutathione; GPX, glutathione peroxidase; GR, glutathione reductase.

GSH, also called L-gamma-glutamyl-L-cysteinyl-glycine, the most abundant antioxidant, plays a central role in the maintenance of redox homeostasis by regulating therapeutic resistance; metabolic reprogramming; and ferroptosis, a form of iron-dependent cell death characterized by lipid peroxidation, by scavenging reactive oxygen species (ROS), detoxifying electrophilic compounds, and stabilizing the cellular redox environment [6,7,8]. GSH is synthesized and regenerated by cystine uptake via the cystine/glutamate antiporter (system Xc^−^) and through an NADPH-dependent reduction of oxidized glutathione (GSSG) by GR [9]. We illustrate GSH-mediated redox regulation and ferroptosis in lung cancer cells in Figure 1. In this section, we highlight the GSH-associated mechanisms and strategies against lung cancers. Moreover, GSH-associated awareness is mentioned.

### 3.2. Nrf2–GSH Axis

Elevated GSH levels are directly associated with resistance to chemotherapeutic agents, such as cisplatin, in A549 NSCLC cells. However, GSH depletion caused by flavonoids, such as 2′,5′-dihydroxychalcone and chrysin, significantly increases cisplatin-induced cytotoxicity in A549 cells [11]. GSH-dependent chemoresistance is regulated by nuclear factor Nrf2, a master antioxidant response regulator. Nrf2 depletion caused by shRNA results in decreased GSH levels, ROS accumulation, and increased sensitivity to ionizing radiation; these effects can be attenuated by pretreatment with antioxidants, such as *N*-acetylcysteine, GSH, and vitamin E, in A549 cells [5].

In addition, the Nrf2–GSH axis is amplified by the linker histone variant H1.2, which interacts with and stabilizes nuclear Nrf2 and sustains GSH synthesis in NSCLC tissues and A549 cells [12]. H1.2 deletion suppresses cancer progression and reduces GSH levels in small GTPase KRas-driven NSCLC mouse models [12], suggesting that the attenuation of the H1.2-Nrf2 axis could be a potential therapeutic strategy against lung cancer progression. Pharmacological inhibition of the Nrf2/Kelch-like enoyl-CoA hydratase-associated protein 1 (Keap1) pathway by K-563, a natural compound derived from *Streptomyces* species, suppresses GSH biosynthesis and elevates intracellular ROS levels in lung cancer cells, suggesting that K-563 is a potential chemotherapeutic agent [13]. Moreover, Nrf2 downregulation via microRNA-365a-3p induces GSH depletion and lipid ROS accumulation in A549 and H1299 cells [14]. Both the genetic and pharmacological disruption of the Nrf2-GSH axis impairs redox homeostasis and promotes cancer cell death, offering a potential strategy for improving treatment efficacy and survival in patients with cisplatin-resistant NSCLC [15]. The detailed mechanism of the Nrf2/Keap1 pathway is discussed in the Nrf2 section.

### 3.3. GSH Depletion Strategy in the Lung Cancer System

Given the central role of GSH in cancer cell survival, several therapeutic strategies have been developed to exploit GSH dependency. Recently, for instance, disulfide-bridged mesoporous organosilica nanoparticles have been found to deplete the GSH pool and release cisplatin, thereby enhancing DNA damage and apoptosis in cisplatin-resistant NSCLC models, both in vitro and in vivo without toxicity [16]. Similarly, the natural compound for the anti-cancer drug steroidal saponin timosaponin AIII (Tim-AIII) induces ferroptosis by directly binding to heat shock protein 90, the facilitation of ubiquitination, and the degradation of GPX4, thereby promoting GSH depletion, lipid peroxidation caused by ROS, and subsequent cell death in A549 and H1299 cells [17]. Briefly, it is known that GPX4 modulates the oxidative activity of lipid peroxides in a GSH-associated manner to protect cells against ferroptosis [18]. Moreover, Tim-AIII prevents cancer growth by enhancing ferroptosis in a subcutaneous NSCLC xenograft model [17].

GSH synthesis also depends on cystine uptake via solute carrier family 7 member 11 (SLC7A11), a critical component of system Xc^−^ [19]. The membrane localization of SLC7A11 is modulated by the cytoskeletal protein spectrin beta, non-erythrocytic 2 (SPTBN2), a ferroptosis suppressor [20]. In contrast, SPTBN2 depletion impairs SLC7A11 trafficking, reduces GSH biosynthesis, and enhances sensitivity to cisplatin and ferroptosis inducers in A549 and H1299 cells [20], suggesting that SPTBN2 could be a therapeutic target for cisplatin resistance. Moreover, the natural compound abrine has been identified as a SPTBN2 inhibitor that potentiates ferroptosis through the mislocalization of SLC7A11 and GSH depletion in A549 cells [20]. The targeting of GSH in lung cancer cells is summarized in Table 1.

### 3.4. GSH Rebound Mediates Potential Awareness

Treatment with fenofibrate, a peroxisome proliferator-activated receptor alpha (PPARα) agonist, attenuates cisplatin cytotoxicity in A549 cells through the activation of the aryl hydrocarbon receptor–Nrf2 axis, which is related to the increased expression of antioxidant enzymes and enhanced GSH synthesis, which protects cancer cells [21]. Thus, co-treatment with fenofibrate and ROS-associated chemotherapeutic agents should be approached cautiously because of the potential for redox-mediated chemoresistance.

## 4. Nrf2

### 4.1. Endogenous Anti-Oxidative Role of Nrf2

Nrf2 is an oxidative-component-associated transcriptional regulator that is associated with the antioxidant defense system. Although we have described Nrf2 in the GSH section and reviewed it in various articles [22,23,24,25], Nrf2 and its associated mechanisms are mentioned in this section. Differences in the oxidative stress defense system have been addressed in several lung cancer cell types, such as Nrf2-enriched A549 cells, compared to NCI-H292 cells [26]. Thus, A549 cells are highly resistant to cisplatin [26]. The constitutive activation of Nrf2 induces enhanced chemoresistance and cancer cell survival through the upregulation of ROS-scavenging systems, such as GSH and TRX [5]. Conversely, RNAi-mediated Nrf2 depletion enhances ROS and protein oxidation, thereby reducing cell survival [5]. The hyperactivation of Nrf2 through mutations in both Keap1 and Nrf2 has been observed in various cancers, such as ovarian cancer and pulmonary papillary adenocarcinoma [27,28]. Knockdown of Keap1 reduces cyclin D1 and cancer stem cell markers, whereas it enhances PPARγ in chemotherapeutic agent arsenic trioxide-administered A549 cells [29], suggesting that Keap1 modulation is associated with the regulation of chemosensitivity.

In addition to its oxidative stress defense role, Nrf2, as a cancer-activating component, is involved in cancer development [30]. Moreover, the higher expression of Nrf2 and multidrug-resistance-associated protein 1 (MRP1) has been observed more in malignant tumors than in adjacent non-tumors [31]. The multidrug-resistant cell line H69AR has shown enhanced Nrf2-antioxidant response element (ARE) pathway and MRP1 protein expression compared to H69 lung cancer cells [31]. Based on an analysis of immunohistochemical data in cancer tissues, we can say that the Nrf2 and antioxidant response element pathways regulate MRP1 expression [31].

As mentioned above, the oxidative stress defense systems vary between types of lung cancer cells, such as Nrf2-enriched A549 cells, compared to NCI-H292 cells [26]. Moreover, the endocannabinoid system (ECS) is differentially expressed in various cancer cell types. Patients with lung squamous cell carcinoma have enhanced ECS levels, which are determined by anandamide, 2-arachidonylglyceriol, and their receptors, such as cannabinoid 1/2 receptors, transient receptor potential vanilloid 1, and G protein-coupled receptor 55, whereas patients with adenocarcinoma show reduced levels of ECS [32]. Thus, antioxidant and cannabinoid systems in various cancer types are simultaneously considered prominent differential factors in the development of anti-cancer therapy.

### 4.2. Nrf2/Keap1-Associated Lung Cancer Therapies

Although we discussed the Nrf2/Keap1 pathway, which is associated with GSH, in Section 3, various approaches to lung cancers have been addressed with respect to the modulation of the Nrf2 or Nrf2/Keap1 axis. For instance, FAM129B, Niban-like protein 1, is a competitive inhibitor of Nrf2 that binds to Keap1 by reducing the ubiquitination of Nrf2 [33]. As mentioned in the GSH section, the pharmacological inhibition of K563 in the Keap1/Nrf2 pathway suppresses Nrf2-associated gene expression, reduces GSH biosynthesis, and enhances ROS levels [13]. Co-treatment with retinoic acid enhances cisplatin sensitivity and suppresses DNA repair through homologous recombination in A549 cells [34]. GTPase KRas is associated with cancer cell malignancy. KRas modulates the expression of the tumor suppressor p53 at low levels [35]. KRas depletion stabilizes p53 and suppresses Nrf2, NAD(P)H quinone dehydrogenase 1 (NQO1), and GSH [35].

In addition, the long noncoding RNA metallothionein 1D pseudogene (MT1DP) has been reported to enhance ferroptosis-mediated cell death in NSCLC cells by modulating the miR-365a-3p/Nrf2 axis [14]. MT1DP stabilizes miR-365a-3p, which, in turn, suppresses Nrf2 expression, leading to reduced GSH levels and elevated lipid ROS, thereby sensitizing lung cancer cells to ferroptosis [14]. In a drug-repurposing approach, the anti-psoriatic drug clobetasol propionate, an Nrf2 inhibitor, induces ferroptosis and iron-mediated ROS accumulation [36]. Radiation combined with clobetasol propionate enhances ferroptic cell death in A549 cells [36]. Moreover, a combined approach using metformin and cisplatin has been used in a NSCLC cell–xenograft model [37]. Metformin strongly inhibits Nrf2 by interacting with ERK1/2 and enhancing proteasomal degradation [37], suggesting that metformin induces the cisplatin-mediated oxidative stress defense system in lung cancer. The lipid-nanoparticle-launched Nrf2 inhibitor quinacrine improves cisplatin-mediated lung cancer cell death through enhanced cell permeabilization and effective Nrf2 downregulation [38]. More recently, the PDZ-binding motif (TAZ) was found to be associated with the Nrf2 signaling pathway in normal lung tissues [39]. TAZ deficiency induces dysregulated Nrf2 signaling, autophagy, and the accumulation of autophagosomes and ROS, subsequently inducing cell death [39]. Although the TAZ-mediated dysregulation of Nrf2 is an attractive therapeutic strategy, cancer-cell-specific regulation is required. Pharmacological and genetic inhibition, or combinational treatments for the Nrf2-associated axis, dysregulates redox regulation and subsequently impairs cancer cell survival. We summarized therapeutic strategies targeting the Nrf2 pathway in lung cancers in Table 2.

## 5. SOD

SOD, a catalytic enzyme, is involved in the conversion of superoxide into oxygen and H_2_O_2_ and prevents cellular damage caused by excess ROS [40].

**Figure 2:**
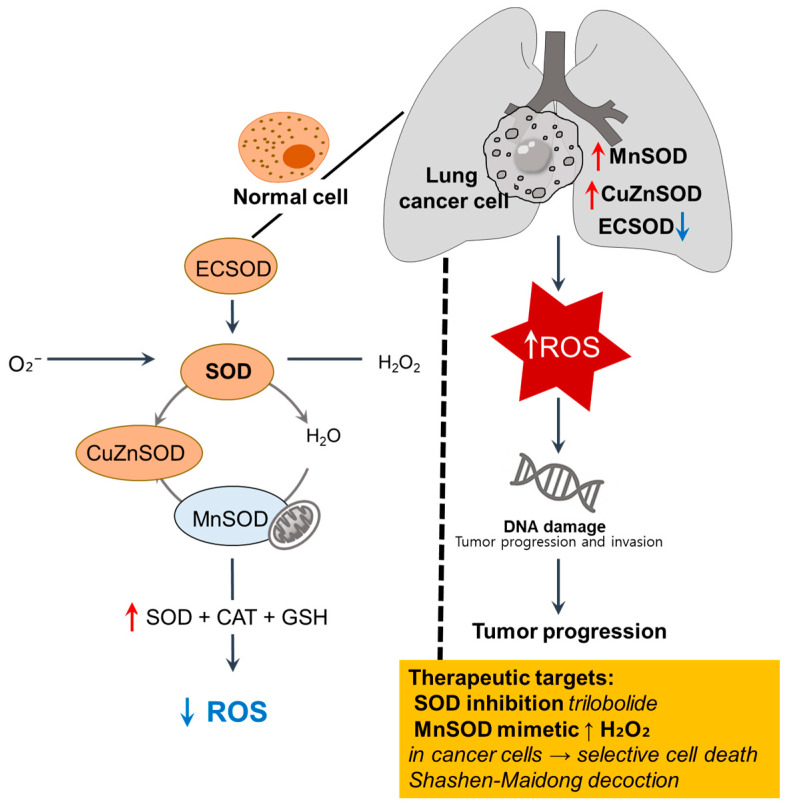
Imbalance of SOD isoforms and redox dysregulation in lung cancer cells. In normal cells, ECSOD, cytosolic Cu/ZnSOD, and mitochondrial MnSOD catalyze the dismutation of superoxide (O_2_^−^) into H_2_O_2_, which is further detoxified by CAT and GSH to maintain redox balance [40]. In lung cancer cells, MnSOD and CuZnSOD are upregulated, whereas ECSOD is downregulated, contributing to ROS accumulation, oxidative DNA damage, and tumor progression [41,42,43]. ↑, upregulation; ↓, downregulation; SOD, superoxide dismutase; ROS, reactive oxygen species; ECSOD, extracellular SOD.

The altered expression and activity of SOD isoforms, manganese SOD (MnSOD), copper/zinc SOD (CuZnSOD), and extracellular SOD (ECSOD) as first-line antioxidant defense components are recurrent features observed in lung cancer, reflecting dysregulated redox homeostasis across tissue, cellular, and systemic levels [41,42]. We illustrate the imbalance of SOD isoforms and redox dysregulation in lung cancer cells in Figure 2.

Erythrocyte SOD activity is significantly reduced in patients with lung cancer compared to that in healthy individuals, with a further decline noted in advanced disease stages, suggesting a systemic antioxidant deficiency associated with cancer progression [41]. Tumor tissues have revealed heterogeneous SOD expression patterns with upregulated MnSOD and CuZnSOD, whereas ECSOD is consistently downregulated, particularly in the extracellular compartment [42].

Notably, high CAT levels have been observed in healthy people, suggesting that dysregulated ROS levels may cause DNA damage and cancer development [43]. Adenocarcinoma A549 cells exhibit tumor necrosis factor-α-mediated MnSOD activity. Moreover, A549 cells contain high CAT activity, high levels of GSH, and γ-glutamylcysteine synthetase immunoreactivity [44], suggesting that differential SOD profiles are associated with variable resistance to oxidative stress and chemotherapeutic agents. The upregulation of MnSOD within tumors likely reflects an intracellular attempt to reduce mitochondrial ROS, whereas diminished ECSOD is involved in facilitating extracellular oxidative signaling, which promotes tumor invasion [42,43].

The traditional herbal formula Shashen-Maidong Decoction suppresses tumor growth under intermittent hypoxia by restoring mitochondrial SOD2 expression and downregulating IL-6/JAK2/STAT3 signaling, thereby linking oxidative and inflammatory responses [45]. Additionally, trilobolide-6-O-isobutyrate, a compound isolated from *Sphagneticola trilobata*, triggers apoptosis-like cell death in NSCLC cells through intracellular ROS accumulation, accompanied by the depletion of GSH and SOD [46]. Furthermore, the combination of pharmacological ascorbate and MnSOD mimetic rucosopasem selectively elevates the H_2_O_2_ level in NSCLC cells and subsequently enhances radio- and chemo-sensitivity without harming normal bronchial epithelial cells [47]. Given the differences between cancer and normal cell oxidative metabolism, newly developed pentaazamacrocyclic Mn (II)-containing (MnPAM) SOD, combined with pharmacological ascorbate, enhances the radiation-mediated therapeutic efficacy in NSCLC cells [47], suggesting that cancerous SODs could be potential therapeutic targets, and the development of targeting strategies for cancerous redox-regulating enzymes should be prominent therapeutic outcomes.

## 6. CAT

**Figure 3:**
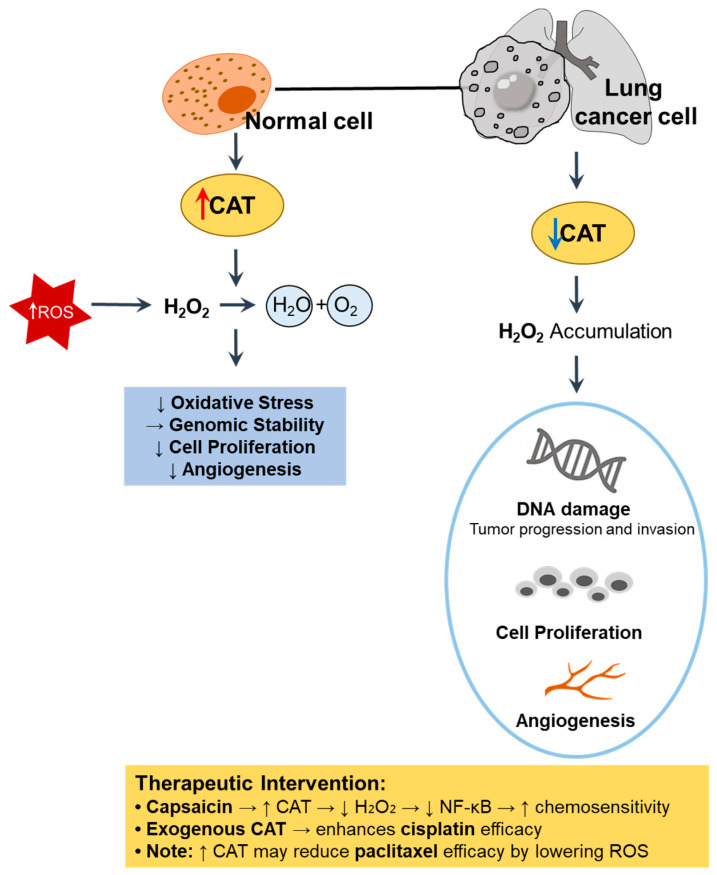
Role of CAT in redox regulation and tumor progression in lung cancer. In normal cells, high CAT activity detoxifies H_2_O_2_, thereby reducing oxidative stress, maintaining genomic stability, and suppressing abnormal proliferation and angiogenesis [48,49]. In contrast, CAT downregulation in lung cancer cells leads to H_2_O_2_ accumulation, DNA damage, and enhanced tumor progression [49,50]. ↑, upregulation; ↓, downregulation. CAT, catalase; ROS, reactive oxygen species.

CAT, a key antioxidant enzyme, is involved in the detoxification of H_2_O_2_ through conversion into water and oxygen [48]. Thus, decreased CAT activity results in the accumulation of H_2_O_2_, which acts as a secondary messenger to promote cell proliferation, DNA damage, and angiogenesis in cancer cells [49]. We illustrate the cellular mechanism and therapeutic intervention of CAT in Figure 3.

CAT activity is reduced or dysregulated in lung cancer cells and tissues. For instance, oral administration of the chemical carcinogen benzo(*a*)pyrene induces lung carcinogenesis in mice through the notable suppression of CAT and other antioxidant enzymes [50]. Treatment with capsaicin, an ingredient of red pepper, restores CAT activity in a benzo(*a*)pyrene-induced lung cancer mouse model [50]. Similarly, CAT activity is significantly decreased in both squamous cell carcinoma and adenocarcinoma tissues compared to adjacent healthy tissues [32]. Thus, CAT downregulation contributes to an oxidative environment that is favorable for lung cancer initiation and progression.

However, modulating CAT expression or exogenous supplementation may enhance therapeutic outcomes by disrupting the redox balance in cancer cells. Regarding the oxidative regulatory role of CAT, exogenous CAT administration produces a cytostatic effect, suppresses NF-κB activation, and enhances the cytotoxicity of several chemotherapeutic agents, most notably cisplatin, in A549 cells, suggesting that the restoration of CAT activity counteracts cancer resistance mechanisms [51]. However, this interaction is drug-specific, as CAT antagonizes paclitaxel efficacy by reducing H_2_O_2_ levels and enhancing cell viability, reflecting the complexity of redox–drug interactions [51]. In a benzo(*a*)pyrene-induced lung cancer model, the upregulation of CAT through hyperoxia combined with carboplatin significantly enhanced apoptosis, indicating a synergistic interaction between oxidative stress and chemotherapy [52]. Similarly, CAT-mimetic platinum nanoparticles have been shown to improve chemotherapeutic outcomes by relieving cancer hypoxia, thereby enhancing drug efficacy [53]. However, CAT reduces H_2_O_2_ levels induced by paclitaxel, thereby interfering with its pro-oxidant anti-cancer effects and promoting cancer cell survival [51]. The overexpression of CAT in NSCLC cells attenuates the cytotoxicity of redox-based radio-chemo-sensitizers, such as rucosopasem manganese and pharmacological ascorbate, by reducing H_2_O_2_ accumulation [47]. These findings collectively highlight the dual role of CAT as both a protective antioxidant and modulator of treatment responses, emphasizing the need for the careful consideration of redox dynamics when integrating CAT-targeted strategies into lung cancer therapy.

## 7. HO-1

HO-1 is a derivative of heme oxygenase and a catalytic enzyme that produces iron, carbon monoxide, and biliverdin (converted into bilirubin) [3,54]. HO-1 possesses antioxidant, antiviral, anti-inflammatory, anti-apoptotic, and neuroprotective effects [54,55,56]. Although HO-1 is essential for maintaining redox homeostasis, its biological effects vary depending on the cellular context and disease stage. In clinical NSCLC samples, tumor-associated macrophages exhibit significantly lower HO-1 expression than macrophages in adjacent non-tumorous lung tissues, indicating impaired local oxidative stress defense [55]. In contrast, the overexpression of Nrf2 and HO-1 in NSCLC cell lines, such as NCI-H292, SK-MES-1, and NCI-H460, promotes the expression of thymidine phosphorylase, IL-6, and IL-1β and subsequently facilitates tumor-associated angiogenesis [57]. HO-1 is involved in lung cancer invasiveness in human patients [58].

Therefore, targeting the HO-1-associated pathway has emerged as a promising therapeutic strategy for the treatment of NSCLC. For instance, propyl gallate, a phenolic compound, significantly enhances the sensitivity of NSCLC cells to cisplatin by reducing HO-1 activity [59]. The combination of metformin and (-)-epigallocatechin-3-gallate increases intracellular ROS levels and induces apoptosis by suppressing both HO-1 and silent information regulator 1 (SIRT1) in A549 cells [60,61]. HO-1 inhibitors have shown promising effects against lung cancer. Treatment with the HO-1 inhibitor, zinc protoporphyrin IX (ZnPPIX), in combination with irradiation, enhances radiosensitivity and induces apoptosis in A549 cells [62]. The noncompetitive inhibitor VP13/47 induces apoptosis and mitochondrial dysfunction by attenuating HO-1 expression and activity in A549 cells [63]. Additionally, overexpressed microRNA-1304 decreases cell viability and induces cell cycle arrest in A549 and H1975 cells [64]. Meanwhile, Smad7 is known as an inhibitory protein of transforming growth factor-beta (TGF-β) signaling and is negatively associated with the Akt/HO-1 survival pathway in cisplatin-administered A549 cells [65], suggesting that Smad7 targeting could be a promising strategy against drug resistance in cancer treatment through HO-1 regulation.

In contrast, garlic oil upregulates HO-1, glutathione S-transferase alpha 1 (GSTA1), and NQO1 and significantly reduces tumor formation in tobacco compound 4-(methylnitrosamino)-1-(3-pyridyl)-1-butanone-exposed mice [66]. Likewise, enhanced HO-1 expression inhibits tumor growth by downregulating matrix metalloproteinases and inflammatory mediators, such as IL-1β, in NCI-H292 mucoepidermoid carcinoma–xenograft models [67]. Collectively, these data indicate that both the upregulation and inhibition of HO-1 yield therapeutic benefits depending on the tumor subtype and redox context.

Recent advances in nanotechnology and nutraceutical intervention have demonstrated the therapeutic versatility of HO-1. Briefly, a nanodrug composed of ferrocene (Fc) and tin protoporphyrin IX suppresses HO-1 activity, elevates intracellular heme levels, and subsequently attenuates NSCLC metastasis [68]. Non-thermal plasma treatment could be considered a potential cancer therapy through the attenuation of the Nrf2/HO-1 axis in H322 and H1299 cells but not in A549 cells [69]. Additionally, inhibition of the histamine N-methyltransferase/human epidermal growth factor receptor 2 axis enhances sensitivity to cisplatin and reduces cancer stem cell properties by disrupting the Nrf2/HO-1 pathway in H441 cells [70]. Nutrients, such as vitamin C (non-enzymatic antioxidant defense system), suppress the metastasis of H22, BEAS-2B, and H1299 cells through HO-1 activation [71]. Similarly, the traditional Chinese herbal formula Yishen Qutong Granules, containing genistein and quercetin, reduces tumor burden by downregulating HO-1 expression in xenograft-bearing mice [72]. Targeting strategies for HO-1-modulation are summarized in Table 3. Collectively, these findings suggest the therapeutic potential of modulating HO-1 activity across a broad spectrum of pharmacological and nutritional platforms for lung cancer treatment.

## 8. PRXs

PRXs are a family of thiol-dependent peroxidases that reduce H_2_O_2_ and organic hydroperoxides, thereby contributing to redox balance and cellular protection. PRX1, PRX2, PRX4, and PRX6 are frequently overexpressed, particularly in lung adenocarcinomas and high-grade squamous cell carcinomas, as demonstrated by immunohistochemical and RT-PCR analyses [73]. Specifically, elevated PRX2 expression correlates with an advanced tumor stage, whereas PRX4 is predominantly expressed in lung adenocarcinomas [73]. A PRX4-overexpressed transgenic mouse model revealed that reduced oxidative stress through overexpressed PRX4 promotes cancer development, such as with highly penetrated microvessels in tumors; upregulated cytokine levels, including IL-1β and matrix metallopeptidase 9; and enhanced tumor size [74]. These findings indicate that PRXs not only function as antioxidants but also contribute to lung tumor proliferation and progression.

## 9. GRXs

GRXs and TRXs are widely distributed disulfide reductase enzymes [75]. Among them, GRXs are crucial regulators of cellular redox homeostasis through catalyzing glutathione-dependent thiol–disulfide exchange reactions [76]. GRXs comprise four principal isoforms. GRX1 and GRX3 are predominantly cytosolic, while GRX5 is mainly mitochondrial [77]. GRX2 exists in multiple forms, including the mitochondrial GRX2a and the cytosolic/nuclear GRX2b and GRX2c [78,79,80]. Notably, GRX3 (also known as PICOT) exhibits significant overexpression in lung cancer tissues compared to normal tissues [77]. In addition, elevated GRX expression has been observed in NSCLC cells resistant to gefitinib, a commonly used epidermal growth factor receptor (EGFR) tyrosine kinase inhibitor [81]. Thus, the inhibition of GRX in gefitinib-resistant NSCLC cells, carrying the EGFR mutation, enhances the efficacy of gefitinib by promoting apoptosis and cell cycle arrest through the modulation of the EGFR/FoxM1 signaling pathway [81]. On an epigenetic level, GRX3 interacts with embryonic ectoderm development protein and regulates the methylation process at the promoter of the *CCND2* gene [82]. Additionally, the radiation-induced TP53-regulated inhibitor of apoptosis 1 (TRIAP1) regulates the expression of GRX2 and GRX3 in NSCLC cells [83]. The knockdown of TRIAP1 disrupts the expression of GRXs and other antioxidant defense components such as TRX and PRXs, leading to increased ROS and enhanced radiosensitivity [83]. Taken together, GRXs contribute to lung cancer progression by modulating redox balance, epigenetic gene regulation, and resistance to both targeted therapies and radiotherapy.

## 10. Thioredoxin and Thioredoxin Reductases

TRX and TrxR consist of a functional redox pair. Basically, TRX donates electrons to reduce protein disulfides, while TrxR uses NADPH to restore TRX to its reduced state [84]. Several strategies that target TRX and TrxR have been developed to treat lung cancer. For instance, PX-12 (1-methylpropyl 2-imidazolyl disulfide), an inhibitor of TRX1, induces an anti-cancer effect through cell cycle arrest in lung cancer cells such as A549 and Calu-6 cells [85,86]. Additionally, TrxR is considered an attractive target for cancer because of its overexpressed feature in cancers [87,88]. The combined application of dimethoxycurcumin, an analog of curcumin, synergistically enhances apoptosis through the inhibition of TrxR and subsequently enhances radiosensitivity in A549 cells [89]. The inhibitor of TrxR shikonin induces ROS-mediated necrosis [90]. Its combined application with BAY876, a glucose transporter inhibitor, enhances cytotoxicity and reduces the resistance of Keap1-mutated NSCLC cells [90]. This effect is attributed to NADPH depletion under glucose-limited conditions, leading to disrupted redox homeostasis and increased ROS accumulation. In addition, the pharmacological inhibition of glucose-6-phosphate dehydrogenase caused by 6-aminonicotinamide (6-AN) further increases shikonin-induced cytotoxicity, albeit without selectivity for Keap1 mutation [90]. In addition, the TrxR inhibitor auranofin induces cell death by enhancing ROS levels and depleting GSH levels in several lung cancer cell lines, including Calu-6 and A549 [91]. As a developed approach to treat cancer, the combined application of auranofin and the tyrosine kinase inhibitor lenvatinib enhances ROS accumulation, endoplasmic reticulum stress, and ROS-dependent JNK signaling and subsequently induces synergistic anti-cancer effects in human lung cancer cells, such as H1299, H520, and A549 cells [92]. Treatment with plumbagin, a hydroxy-1,4-naphthoquinone, produces ROS in NSCLC cells [93]. However, Nrf2-constitutive active lung cancer cells are resistant to plumbagin. Thus, the combined application of plumbagin and BAY876 or 6-AN induces synergistic apoptotic effects and overcomes plumbagin resistance in Keap1-mutant NSCLC cells [94]. Recently, TrxR inhibitors have been developed and optimized. For instance, thimerosal has been identified as a strong TrxR inhibitor [95]. LW-216 also inhibits TrxR by binding at R371 and G442 [96]. Treatment with LW-216 reduces TrxR expression and reveals an anti-cancer effect on NSCLC-implanted mice [96]. In addition, the covalently bonded prodrug 5u, which binds to residues of TrxR, C475, and selenocysteine (SeC) 498, mediates the cytotoxicity of NSCLC cells through dysregulated redox modulation and induces apoptosis and ferroptosis [97]. The regulatory strategies targeting TrxR are summarized in Table 4.

For the regulation of GPX, ebselen, a selenoorganic compound, possesses GPX-like activity [98]. Treatment with ebselen induces mitochondrial dysfunction and GSH depletion and subsequently enhances cell cycle arrest and apoptosis in Calu-6 and A549 cells [99]. Accordingly, the development of strategies targeting TrxR inhibitors and the regulation of GPX based on recent studies remain challenging issues.

## 11. 8-oxodG

8-oxodG is a naturally driven mutagenic DNA metabolite used to protect tissues from DNA damage through the transversion of guanine into thymine during DNA replication [100]. Various studies have shown that 8-oxodG is an oxidative stress marker involved in anti-inflammation [101,102,103]. Moreover, the repair mechanism of 8-oxodG is dysregulated in cancer cells. The depletion of 8-oxodG repair often results in genomic alterations in cancer and is found in patients with cancers, including small-cell lung cancer [104,105,106].

Lung tissue is easily exposed to diesel exhaust particles (DPs). High doses or repeated exposure to DPs mediates the production of the oxidative stress marker 8-oxodG [107]. On the other hand, 8-oxodG is considered an antioxidant that scavenges ROS. For instance, 8-oxodG exhibits antioxidant and anti-metastatic properties by inhibiting Rho-GTPase signaling in pancreatic cancer cells [108]. The application of 8-oxodG to oxidative stress-stimulated cardiomyocytes reduces ROS levels and protects cardiac tissue against oxidative damage [109]. Thus, the production of 8-oxodG in damaged cells could be a potential metabolite caused by protective processes against oxidative stress in addition to oxidative stress markers. The verification of the anti-oxidative role of 8-oxodG would be a promising developmental strategy for lung cancers, as it could be used as an endogenous antioxidant defense system.

## 12. Mitochondrial Citrate Carrier (SLC25A1)

SLC25A1 is involved in the oxidative stress defense system for survival in glioblastoma; prostate cancer; and lung cancer cells, such as NCI-H460 and A549 [110]. Oxidative stress stimulation induces the enhanced expression of SLC25A1, whereas treatment with the inhibitor 1,2,3-benzene-tricarboxylic acid reveals dysregulated mitochondrial redox regulation in lung cancer cells [110]. The co-administration of ionizing radiation with an SLC25A1 inhibitor effectively overcomes the enhanced radioresistance of cancer cells [110]. Conversely, SLC25A1 overexpression has been observed in patients with malignant tumors. [110]. SLC25A1 is involved in the mitochondrial citrate pool and redox homeostasis in cancer stem cells [111]. Thus, the inhibition of SLC25A1 induces the dysregulated self-renewal of cancer stem cells [111]. SLC25A1 inhibition caused by citrate transporter protein inhibitor 2 induces D-2-hydroxyglutarate accumulation and delays radiation-induced DNA repair [112]. Despite the precise role of D-2-hydroxyglutarate in cancer cell damage, the inhibition of SLC25A1 has a supportive effect on radiation therapy. Collectively, the modulation of SLC25A1 could be a potential therapeutic strategy against cancer.

## 13. Summary and Perspectives

In this review, we mainly highlighted the enzymatic antioxidant defense module in lung cancers. The enzymatic antioxidant proteins and their regulatory factors—including GSH, Nrf2, SOD, CAT, HO-1, PRXs, GRXs, TRX, TrxR, GPXs, and SLC25A1—described above constitute an organically interconnected network that maintains cellular redox homeostasis [110,111,113]. Briefly, during oxidative phosphorylation, superoxide anions are produced as primary ROS, which are rapidly converted into H_2_O_2_ through the action of SOD [114]. Subsequently, H_2_O_2_ is detoxified by CAT, GPXs, and PRXs, thereby preventing the accumulation of ROS [115,116]. These enzymatic antioxidant systems critically depend on reduced GSH, whose regeneration is mediated by the GRX and TRX systems [84]. In this context, TRX is maintained in its reduced form by TrxR through an NADPH-dependent mechanism [84,117]. Moreover, the expression of numerous antioxidant enzymes, including SOD, CAT, GPX, HO-1, PRX, GRX, and TrxR, is under the transcriptional control of Nrf2 in various systems [15,118,119]. Thus, although we discussed each antioxidant component in this review, the mechanistic interconnection of antioxidant components should be considered a coordinated oxidative stress defense module.

Various approaches using antioxidants against cancers have been developed through the modulation of the antioxidant defense system and the dysregulation of the redox balance. Prominent approaches using antioxidant drugs are attractive for the induction of cancer cell death. However, the goal of antioxidant drug application should be considered a strategy to modify drug efficacy and attenuate the oxidative stress defense system in cancer systems, even in lung cancers. Thus, to develop a strategy for cancer treatment, it is necessary to understand and verify the oxidative stress defense systems of cancer cells. In addition to the enzymatic antioxidant defense components, other non-enzymatic antioxidant defense components should be highlighted in coming years. Moreover, for redox regulation, we provide substantial aspects of these anti-cancer strategies.

### 13.1. Combination Therapies

Combination strategies involving antioxidant regulation with immunotherapy, radiation, or metabolism-targeted therapy should be considered to expand therapeutic potential. Cancer cells are influenced by the oxidative microenvironment, and their interactions with immune cells and stromal components may contribute to redox-associated treatment responses. For example, oxidative stress modulates immune checkpoint expression, potentially affecting the efficacy of immunotherapy [120]. Moreover, the redox state impacts radiation sensitivity by influencing DNA repair and apoptotic pathways. However, compensatory mechanisms against impaired redox systems may destabilize therapeutic efficacy. Therefore, the effective regulation of these compensatory responses through drug combinations represents an important challenge and opportunity for future research. A better understanding of the interplay between redox balance and the tumor microenvironment may inform strategies for optimizing the timing and composition of combination therapies.

### 13.2. Biomarkers and Patient Stratification

Patient-specific redox profiles and the expression of antioxidant-related genes, such as Nrf2 and Keap1, may serve as potential biomarkers to improve therapeutic efficacy and drug sensitivity [17,18]. In addition, mitochondrial redox-associated metabolic regulators, such as the citrate carrier SLC25A1, are considered promising targets for redox-based treatment strategies [110,111]. As described in the SLC25A1 section, the inhibition of SLC25A1 disrupts redox balance and enhances the sensitivity of cancer cells to radiation, suggesting potential value as both a therapeutic target and a stratification marker. In this context, the application of omics-based analyses—such as transcriptomics, metabolomics, and proteomics—could be instrumental in identifying redox-regulating components and discovering novel drug targets and biomarkers. Furthermore, integrating omics data with patient stratification approaches [121] might enable the identification of specific patient subgroups that are more likely to benefit from redox-targeted therapies. For instance, patients with elevated SLC25A1 or dysregulated Nrf2 activity may show heightened sensitivity to redox-modulating agents or radiation. Stratifying cancer patients based on the dataset of the antioxidant defense module, redox status, or metabolic signatures may help optimize treatment efficacy and support the development of personalized therapeutic strategies in lung cancer.

### 13.3. Drug Delivery Innovations

Advances in drug delivery systems—such as nanoparticle-based carriers, liposomes, exosomes, and stimulus-responsive delivery platforms—provide new opportunities to enhance the specificity and efficiency of redox-modulating agents against cancers. These innovations could allow for targeted delivery to cancer cells, reduce systemic toxicity, and support the co-delivery of synergistic agents (e.g., redox-active drugs and radiation-sensitive drugs). The development of redox-responsive drug delivery systems, including ROS-sensitive nano-carriers [122,123] and GSH-responsive vesicles [124], is an attractive strategy to increase cancer-targeting capabilities and minimize damage to normal cells. Moreover, combining patient stratification with innovative drug delivery technologies could enable more precise, more effective, and safer treatment modalities for lung cancer patients. For instance, patients identified through omics-based profiling as possessing high oxidative stress or redox-related transporter expression could be matched with redox-responsive nano-therapeutics for improved outcomes against cancers.

## Figures and Tables

**Table 1:** Recent therapeutic strategies targeting GSH regulation in lung cancers.

Strategies	Mechanisms	Outcomes	Experimental Models	Ref.
Disulfide-bridged organosilica NPs	↓ GSH, ↑ Cisplatin release	↑ DNA damage, ↑ apoptosis	A549/DDP, xenograft	[16]
Tim-AIII	↓ GPX4, ↓GSH, ↑ lipid peroxidation	↑ Ferroptosis, ↓ cancer growth	A549, H1299, xenograft	[17]
SPTBN2 loss/Abrine	↑ SLC7A11 mislocalization, ↓ GSH	↑ Drug sensitivity, ↑ ferroptosis	A549, H358, H1299	[20]

Notes: Abbreviations: A549/DDP, cisplatin-resistant A549 lung cancer cell line (cisplatin = DDP, *cis*-diamminedichloroplatinum (II)); GSH, glutathione; GPX4, glutathione peroxidase 4; H1299, human non-small-cell lung carcinoma cell line; H358, human bronchioalveolar carcinoma cell line; NP, nanoparticle; SLC7A11, solute carrier family 7 member 11 (cystine/glutamate antiporter); SPTBN2, spectrin beta, non-erythrocytic 2; ↑, increase; ↓, decrease.

**Table 2:** Therapeutic strategies targeting Nrf2 pathway in lung cancers.

Strategies and Targets	Nrf2-Related Pathway	Redox Regulation	Effects	Ref.
FAM129B	↓ Ubiquitination (via Keap1)	–	↑ Redox balance	[33]
K563	↓ Nrf2 gene expression	↑ ROS, ↓ GSH	↑ Oxidative stress sensitivity	[13]
Retinoic acid + cisplatin	↓ DNA repair support (via Nrf2)	↑ ROS	↑ Cisplatin sensitivity	[34]
KRas depletion	↓ Nrf2, ↓ NQO1	↓ GSH	↓ Tumor survival	[35]
MT1DP/miR-365a-3p	↓ Nrf2 mRNA	↑ ROS	↑ Ferroptosis in NSCLC	[14]
Clobetasol + Radiation	↓ Nrf2	↑ ROS, ↑ Fe^2+^	↑ Ferroptosis, ↑ cytotoxicity	[36]
Metformin + Cisplatin	↓ Nrf2 (via ERK1/2 degradation)	↑ ROS	↓ Antioxidant defense	[37]
Quinacrine (via LNPs)	↓ Nrf2 signaling	↑ ROS	↑ Cisplatin efficacy	[38]
TAZ (loss of function)	↑ Nrf2 dysregulation	↑ ROS, ↑ Autophagy	↑ Cell damage and death	[39]

Notes: Abbreviations: ERK1/2, extracellular signal-regulated kinase 1/2; FAM129B, family with sequence similarity 129 member B; Fe^2+^, ferrous ion; Keap1, Kelch-like ECH-associated protein 1; LNPs, lipid nanoparticles; miR, microRNA; MT1DP, metallothionein 1D pseudogene; NQO1, NAD(P)H quinone dehydrogenase 1; ROS, reactive oxygen species; TAZ, transcriptional coactivator with PDZ-binding motif; ↑, increase; ↓, decrease.

**Table 3:** Therapeutic strategies targeting HO-1 modulation in lung cancers.

Strategy	Mechanism	Outcome	Experimental Models	Ref.
Propyl gallate	↓ HO-1	↑ Cisplatin sensitivity, ↑ Apoptvvosis	A549	[59]
Metformin + EGCG	↓ HO-1, ↓ SIRT1	↑ ROS, ↑ apoptosis	A549	[60,61]
ZnPPIX + irradiation	↓ HO-1	↑ Radiosensitivity, ↑ apoptosis	A549	[62]
VP13/47	↓ HO-1 activity	↑ Apoptosis, ↑ mitochondrial dysfunction	A549	[63]
miR-1304 overexpression	↓ HO-1	↓ Viability, ↑ cell cycle arrest	A549, H1975	[64]
Smad7 activation	↓ HO-1/Akt	↑ Cisplatin sensitivity	A549	[65]
Garlic oil	↑ HO-1, ↑ GSTA1, ↑ NQO1	↓ Tumor formation	NNK-induced A/J mice	[66]
HO-1 overexpression	↑ HO-1	↓ MMPs, ↓ inflammation, ↓ tumor growth	NCI-H292 xenograft	[67]
TinPPIX/Fc nanodrug	↓ HO-1, ↑ Heme	↓ Metastasis via Bach1 degradation	A549, SCID mouse xenograft	[68]
NTP + ZnPPIX	↓ HO-1 via Nrf2	↑ ROS, ↑ apoptosis	A549, H322, H1299	[69]
HNMT inhibition	↓ HO-1/HER2 axis	↑ Cisplatin sensitivity, ↓ CSC properties	H441	[70]
Vitamin C	↑ HO-1	↓ Metastasis (p53-related ROS)	H22, BEAS-2B, H1299, xenograft	[71]
Yishen Qutong Granules	↓ HO-1	↓ Tumor burden	NSCLC xenograft-bearing mice	[72]

Notes: Abbreviations: CSC, cancer stem cell; Fc, ferrocene; GSTA1, glutathione S-transferase alpha 1; NQO1: NAD(P)H quinone dehydrogenase 1; HER2, human epidermal growth factor receptor 2; HNMT, histamine N-methyltransferase; HO-1, heme oxygenase-1; MMPs, matrix metalloproteinases; NNK, 4-(methylnitrosamino)-1-(3-pyridyl)-1-butanone; NTP, non-thermal plasma; p53, tumor protein p53; SIRT1, silent information regulator 1; SCID, Severe Combined Immunodeficiency; TinPPIX, tin protoporphyrin IX; ZnPPIX, zinc protoporphyrin IX; ↑, increase; ↓, decrease.

**Table 4:** Strategies targeting thioredoxin reductase (TrxR) in lung cancers.

Strategies	Mechanisms	Outcomes	Experimental Models	Ref.
Dimethoxycurcumin + radiation	TrxR1 inhibition, ↑ ROS, ↓ GSH/GSSG	↑ Apoptosis, ↑ radiosensitivity	A549	[89]
Shikonin + BAY876/6-AN	TrxR1 (SeC498) inhibition, ↓ NADPH	↑ Necroptosis, ↓ drug resistance	Keap1-mutant NSCLC	[90]
Auranofin	TrxR1 inhibition, ↑ ROS, ↓ GSH	↑ Cell death, ↓ MMP	Calu-6, A549, H1299	[91]
Auranofin + Lenvatinib	TrxR1 inhibition, ↑ ER stress, ↑ JNK	↑ Synergistic effect	H1299, H520, A549	[92]
Plumbagin	TrxR1 modification, ↑ ROS	↑ Apoptosis, redox imbalance	NSCLC cells, xenograft	[93,94]
Plumbagin + BAY876 /6-AN	TrxR1 modification, ↑ ROS	↑ Apoptosis, ↓ resistance	Keap1-mutant NSCLC	[94]
Thimerosal	TrxR1 inhibition, ↑ ROS	↑ Apoptosis	A549	[95]
LW-216	TrxR1 binding (R371, G442)	↑ TrxR1, ↑ DNA damage	NSCLC mouse model	[96]
5u (Covalent prodrug)	Covalent binding (C475, SeC498)	↑ Redox imbalance, apoptosis, ferroptosis	NSCLC cells, xenograft	[97]

Notes: Abbreviations: 6-AN, 6-aminonicotinamide; C475, cysteine 475; G442, glycine 442; GSSG, glutathione disulfide; JNK, c-Jun N-terminal kinase; MMP, mitochondrial membrane potential; NADPH, nicotinamide adenine dinucleotide phosphate; R371, arginine 371; SeC, selenocysteine; TrxR1, thioredoxin reductase 1; ↑, increase; ↓, decrease.
